# Time to traumatic intracranial hematoma evacuation: contemporary standard and room for improvement

**DOI:** 10.1007/s00068-024-02573-0

**Published:** 2024-06-18

**Authors:** Michael P. Merakis, Natasha Weaver, Angela Fischer, Zsolt J. Balogh

**Affiliations:** John Hunter Hospital & University of Newcastle, Newcastle, NSW Australia

**Keywords:** Level of evidence, Study type: 3b, Retrospective cohort study

## Abstract

**Purpose:**

Traumatic intracranial hematoma (TICH) is a neurosurgical emergency with high mortality and morbidity. The time to operative decompression is a modifiable but inconsistently reported risk factor for TICH patients? outcomes. We aimed to provide contemporary time to evacuation data and long-term trends in timing of TICH evacuation in a trauma system.

**Methods:**

A 13-year retrospective cohort study ending in 2021 at a trauma system with one level-1 trauma center included all patients undergoing urgent craniotomy or craniectomy for evacuation of TICH. Demographics, injury severity and key timeframes of care were collected. Subgroups analyzed were polytrauma versus isolated head injury, direct admissions versus transfers and those who survived versus those who died. Linear regression of times from injury to operating room was performed.

**Results:**

Seventy-eight TICH patients (Age: 35 (22-56); 58 (74%) males; ISS: 25(25-41); AIS head: 5 (4-5); mortality: 21 (27%) patients) were identified. Initial GCS was 8 (3.25-14) which decreased to 3 (3-7) by arrival in the trauma center. There were 46 (59%) patients intubated prior to arrival. Median time from injury to operation was 4.88 (3.63-6.80) hours. Linear regression of injury to OR showed increasing times to operative intervention for direct admissions to the trauma center over the study period (p=0.04). There was no associated change in mortality or Glasgow outcome score over the same time.

**Conclusion:**

This contemporary data shows timing from injury to evacuation is approaching 5 hours. Over the 13-year study period the time to operative intervention significantly increased for direct admissions. This study will guide our institutions response to TICH presentations in the future. Other trauma systems should critically appraise their results with the same reporting standard.

## Background

Traumatic brain injury has a high mortality rate and can leave those who survive profoundly disabled. The national costs of acute inpatient and rehabilitation care adds up to hundreds of millions of dollars per year [1–3]. Global costs are far higher with an estimated 50 million TBI episodes each year totaling $US 400 billion [4].

Outcomes of patients suffering from traumatic intracranial hematoma (TICH) may be improved by decreasing the time from initial injury to operative decompression. There is limited contemporary evidence available about the time to intervention (craniotomy or craniectomy) in patients with subdural or extradural hemorrhage for evacuation of the hematoma. The median time from injury to operating room (OR) in acute intracranial hematoma (OR) reported in the literature ranged from 3 to 7.1 h [5, 6]. Articles published on the topic more than 30 years ago found a positive correlation with decreasing time to surgery and mortality [7–9], other studies, also more than a decade old, have shown negative correlations and no correlation [5, 10]. There are currently no evidence-based standards for the timing of evacuation of traumatic intracranial hematoma.

The time to operative decompression is essential in minimizing secondary injury to brain tissue. Multiple factors such as time from injury to emergency department (ED), time from emergency to first computed tomography (CT) scan of the head and time from CT scan to operating room (OR) contribute to time from injury to OR [8, 11–14]. 

The study aims are to determine the time from injury to OR in TICH patients and the change in it over the study period. Our hypothesis is that the time between injury to evacuation would decrease over the study period.

## Methods

### Patients

The prospectively maintained institutional trauma registry of a level 1 trauma center was queried for patients undergoing craniotomy or craniectomy between 2009 and 2021. General criteria to perform craniectomy or craniotomy to evacuate TICH at our institution are > 1 cm maximal thickness or greater than 0.5 cm of midline shift or focal or global neurological deficits including decreased GCS, pupillary abnormalities, change in speech, motor function or sensorium as compared to the patient’s baseline. Patients were excluded if they had penetrating head injury, underwent non-operative management (non-operative management defined as a decision not to proceed to theatre based on the initial CT scan of the patient’s head), initial non-operative management with subsequent deterioration requiring surgical intervention, if they were coded to have an abbreviated injury score (AIS) head of 6, underwent insertion of intracranial pressure monitoring devices or cerebrospinal fluid diversion therapies only, had a decompressive craniectomy for intracranial pressure (ICP) reduction only or had a repeat CT scan prior to OR. Where medical records were unavailable patients were excluded.

### Data extraction

The trauma registry and the medical records were reviewed after obtaining ethics approval from the institution’s ethics committee on September 13, 2022. (Approval number: AU202209-07). Patients’ demographic data, injury severity details and outcome data were extracted. Patient’s age, sex, injury severity score (ISS), Abbreviated injury score head (AIS), Glasgow coma scale (GCS) on ambulance arrival and GCS on ED arrival and intubation status were recorded. Computed tomography (CT) head reports were reviewed to determine the type of hematoma, rates of depressed skull fracture and herniation pattern. Operative data was recorded from operation, anesthetic and perioperative reports including type of operation performed.

Times of key events were obtained from ambulance reports, radiology reports, operation, anesthetic and perioperative reports, and discharge summaries. The time and date of initial injury (defined as the bystander’s report of the exact time of injury if documented in the ambulance notes or as the first contact time documented by pre-hospital emergency services), call to emergency services, arrival of emergency services, scene left by emergency services, arrival to emergency department (both peripheral site and tertiary ED arrival), time emergency department left (both peripheral site and tertiary ED arrival), initial CT head, operation start time, operation ending time, time and date of discharge from ICU and from hospital were recorded. Using the time points above, the difference in times between key events were calculated to show the duration between events.

### Statistical methods

Descriptive statistics were summarized for the total sample and separately for subgroups of interest. Subgroups of interest were polytrauma (defined as AIS ≥ 3 in at least 2 body regions) versus isolated head injury and direct admissions versus referrals from peripheral hospitals. Categorical variables were summarized using frequency count and percentage. Continuous variables were summarized by median and interquartile range (IQR). Differences between groups for continuous variables were analyzed using the Kruskal-Wallis test. Differences between groups for categorical variables were tested using the χ^2^ test.

Time in hours from injury to operation was plotted against date of the injury as a scatterplot with linear fit. Linear regression analysis was used to test whether the time from injury to operation changed on average over the study’s period. Results were presented as slope estimates with 95% confidence intervals and associated p-values. Residual diagnostic plots were used to assess the validity of the linear model assumptions. Outliers were identified via a ROUT analysis with Q = 0.5%. A sensitivity analysis was conducted to determine the effect of removing an outlier on the slope estimate.

Data was analyzed using JASP Version 0.16.4 (JASP Team, 2022, RRID: SCR_015823) and Prism 9 for macOS (Version 9.5.0 (525), RRID: SCR_002798).

## Results

**Fig. 1:**
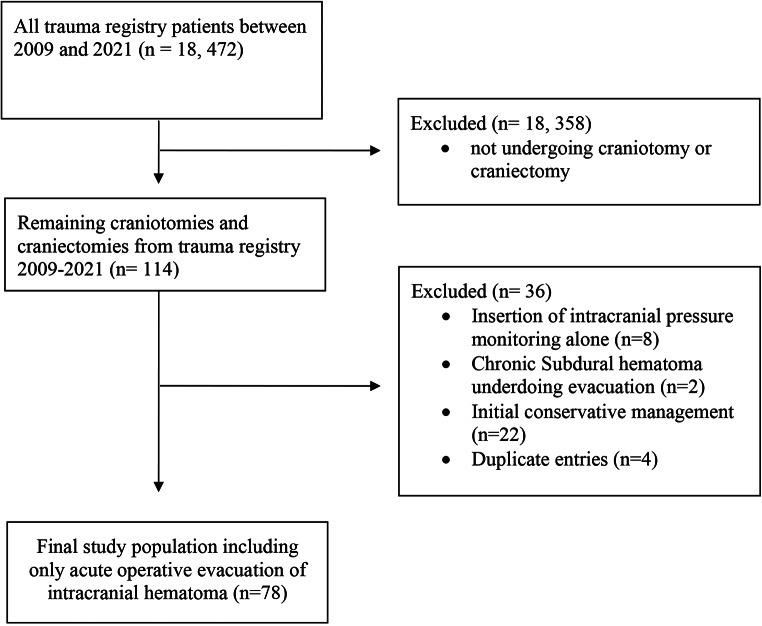
Study inclusion diagram

There were 18, 472 patients identified in the trauma registry between 2009 and 2021, patients not underdoing craniotomy or craniectomy were excluded (*n* = 18,358). There were 114 potentially eligible craniotomy and craniectomy patients identified. Patients were excluded if they received ICP monitoring alone (*n* = 8), chronic hematoma evacuation (*n* = 2), initial conservative management without an immediate operation or had a repeat CT scan prior to OR (*n* = 21). Duplicate entries for operations on the same patient were excluded (*n* = 4). The final study sample included 78 patients (Fig. 1).

**Fig. 2:**
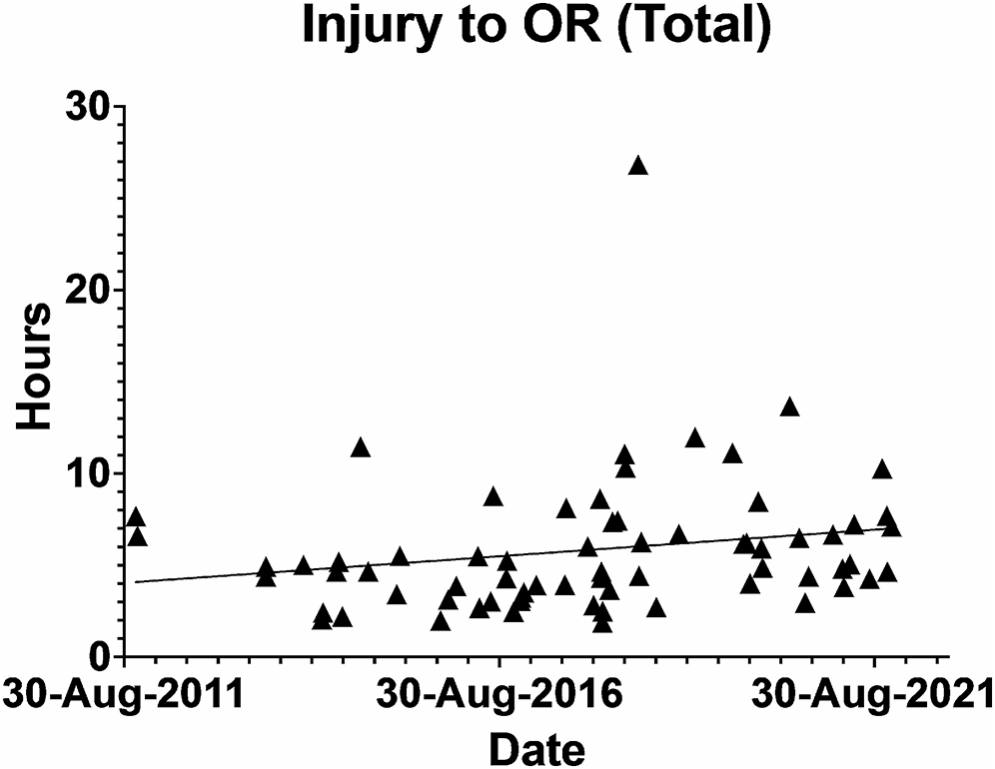
Injury to OR (Total)

Median age was 35 (IQR 22–56), 58 (74%) were male, ISS was 25 (25–41) and AIS head, 5 (4–5). GCS on scene was 8 (3.25-14) and decreased to 3 (3–7) by the time patients had arrived in ED for the whole cohort. Patient undergoing pre-hospital intubated had a median GCS of 7 (3–12) which decreased to 3 (3–3) by ED presentation. Those not underdoing pre-hospital intubation had a median pre-hospital GCS of 13 (4–15) which had decreased to 10 (3–14) on arrival to ED. Forty-six (59%) patients were intubated prior to arrival in ED. CT reports showed that 26 (34%) had a pattern of herniation present, 12 (16%) had a depressed skull fracture, 28 (36%) had an extradural hematoma (EDH), 33 (42%) had a subdural hematoma (SDH), the remaining 17 (22%) suffered from a mixed hemorrhage type (Table 1 and Fig. 2).

**Table 1:** Baseline demographics

	Total (n = 78)	IHI (n = 37)	PT (n = 41)	P values	Direct Admissions (n = 41)	Referrals (n = 25)	P values
Age (years)	35 (22–56)	41 (21.75–56.25)	34 (23–53)	0.65	45 (25–61)	26 (17.5–36.5)	0.002
Sex (male)	58 (74%)	28 (76%)	30 (73%)	0.86	41 (77%)	17 (68%)	0.32
ISS	25 (25–41)	25 (16–25)	41 (32–48)	< 0.001	25 (25–41)	25 (25–41)	0.83
AIS Head	5 (4–5)	5 (4–5)	5 (4–5)	0.14	5 (4–5)	5 (4–5)	0.25
GCS on scene	8 (3.25-14)	7 (3.25-15)	8 (3.75-13)	0.59	6 (3–13)	12.5 (7.5–15)	0.01
GCS in ED	3 (3–7)	3 (3-10.5)	3 (3–7)	0.17	3 (3–8)	3 (3–4)	0.48
Pre-hospital intubation	46 (59%)	17 (46%)	29 (70%)	0.04	26 (49%)	20 (80%)	0.004
Herniation present	26 (34%)	16 (44%)	10 (24%)	0.09	19 (36%)	7 (29%)	0.41
Depressed skull fracture present	12 (16%)	2 (5.56%)	10 (24%)	0.03	10 (19%)	2 (8.33%)	0.26
Hematoma type							
Extradural hematoma	28 (36%)	14 (38%)	14 (34%)	0.76	17 (32%)	11 (44%)	0.31
Subdural hematoma	33 (42%)	17 (46%)	16 (39%)	0.62	25 (47%)	8. 32%)	0.21
Mixed hematoma	17 (22%)	6 (16%)	11 (27%)	0.35	11 (21%)	6 (24%)	0.75

The median duration between ambulance arrival to the scene and arrival to an emergency department was 1.23 (0.89–2.15) hours. The pre-trauma center time was 1.83 (1.30–4.02) hours. The median injury to operation time was 4.88 (3.63–6.80) hours, injury to CT was 3.08 (2.23–3.71) hours and CT to OR was 1.46 (0.70–3.44) hours.

The median length of stay in ICU was 7 (2–12) days while median total length of stay was 14 (5–43) days. The overall mortality rate was 27% (*n* = 21) (Table 2).

**Table 2:** Timing data (hours)

	Total (n = 78)	IHI (n = 37)	PT (n = 41)	P values	Direct Admissions (n = 41)	Referrals (n = 25)	P values
Injury to Ambulance	0.32 (0.18–0.68)	0.28 (0.17–0.45)	0.42 (0.23–0.82)	0.12	0.28 (0.18–0.53)	0.94 (0.45–1.31)	0.03
Ambulance to ED	1.23 (0.89–2.15)	1.17 (0.66–1.60)	1.28 (1.05–2.30)	< 0.001	1.15 (0.82–1.52)	2.83 (2.33–5.74)	0.16
Pre-hospital (Scene time)	0.42 (0.28–0.75)	0.4 (0.33–0.68)	0.5 (0.28–0.79)	0.55	0.4 (0.28–0.68)	0.77 (4.40–8.27)	< 0.001
Pre-hospital time, Injury to tertiary referral ED (for all patients)	1.83 (1.30–4.02)	1.77 (1.25–4.05)	1.83 (1.30–3.67)	0.74	1.52 (1.17–2.19)	6.1 (4.40–8.27)	< 0.001
ED time (ED arrival to exit)	1.6 (0.93–2.37)	1.43 (0.96–2.10)	1.98 (0.91–2.61)	0.52	1.89 (1.15–2.47)	0.87 (0.42–1.55)	0.01
Injury to CT Head	3.08 (2.23–3.71)	2.95 (2.92–2.20)	3.21 (2.64–3.82)	0.46	3.05 (2.38–3.40)	3.95 (2.22–4.83)	0.22
ED arrival to CT Head	1.2 (0.91–1.74)	1.05 (0.89–1.50)	1.47 (0.98–1.90)	0.05	1.22 (0.92–1.74)	1.07 (0.87–1.60)	0.72
Injury to operating room	4.88 (3.63–6.80)	4.43 (3.27–7.20)	5.38 (4.20–6.68)	0.25	4.37 (3.17–5.51)	8.12 (6.08–9.91)	< 0.001
ED arrival to operating room	2.1 (1.21–3.15)	1.97 (1.28–2.94)	2.45 (1.16–3.30)	0.71	2.64 (1.66–3.53)	0.97 (0.60–1.96)	< 0.001
CT head to operating room	1.46 (0.70–3.44)	1.26 (0.61–1.76)	2.02 (0.75–3.95)	0.28	1.09 (0.65–1.87)	5.01 (1.46–7.03)	< 0.001

Injury to operating room time was significantly shorter for direct admissions than it was for patients referred from a peripheral hospital (4.37 versus 8.12 h, *p* < 0.001). ED arrival to OR time was shorter in those who had been referred from a peripheral hospital (0.97 h) versus direct admissions (2.64 h), *p* < 0.001. CT head to OR time was shorter for direct admissions (1.09 h) as compared to referred patients (5.01 h), *p* < 0.001.

Injury to operating room for those who died versus those survived was not statistically different (4.07 (3.23–5.28) hours on average versus 5.22 (4.00-7.15) respectively, *p* = 0.21). Injury to operating room times were longer for those with a favourable neurological outcome as compared to those with a poor outcome. (5.78 (4.42–7.28) hours versus 3.87 (3.02–5.38) hours, *p* = 0.03) (Table 3).

**Table 3:** Durations and LOS, outcomes

	Total (n = 78)	IHI (n = 37)	PT (n = 41)	P values	Direct Admissions (n = 41)	Referrals (n = 25)	P values
Duration of operation (hours)	1.83 (1.47–2.18)	1.9 (1.43–2.41)	1.8 (1.48–2.07)	0.54	1.83 (1.46–2.17)	1.67 (1.39–2.17)	0.58
ICU Length of stay (days)	7 (2–12)	5 (2–11)	8 (3–15)	0.08	8 (2–13)	6 (2–10)	0.81
Length of stay total (days)	14 (5–43)	10 (4–31)	16 (7–54)	0.16	13 (5–48)	16 (5–28)	0.96
Mortality (%)	21 (27%)	11 (30%)	10 (24%)	0.60	19 (36%)	2 (8.0%)	< 0.001
GOS Score @ discharge	4 (1–5)	4 (1–5)	4 (3–5)	0.66	3 (1–4)	4 (4–5)	< 0.001
GOS Score @ follow up 6 + months	4 (1–5)	4 (1–5)	4 (3–5)	0.64	4 (1–5)	5 (4–5)	< 0.001

NB: Categorical variables are summarised with count and a percentage, presented as: Count (Percent %), p values are generated from χ^2^ test. Continuous variables were summarised using median and are presented as: Median (Interquartile range), p values are generated with ANOVA testing using a Kruskal-Wallis test

Abbreviations: Isolated head injury (IHI), Polytrauma (PT), Glasgow coma scale (GCS), Abbreviated injury score (AIS), CT (computed tomography), Intensive care unit (ICU), ED (Emergency department), Glasgow outcome score (GOS)

### Longitudinal results

**Fig. 3:**
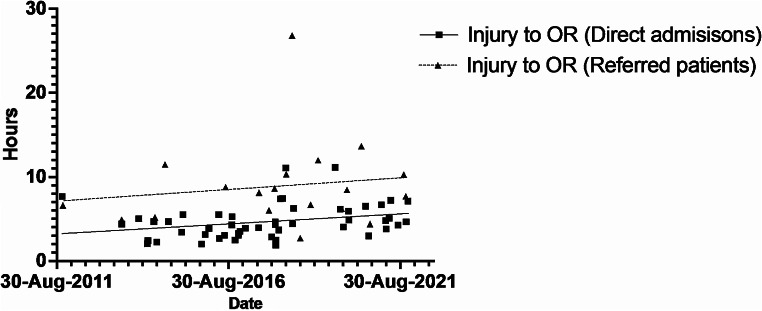
Injury to OR (Direct admissions versus referred patients)

Linear regression results showed that time from injury to operating room increased on average over the study period for direct admissions (slope of trend line = 0.24 [0.01–0.47], *p* = 0.04) (Fig. 3; Table 4). The slope of the trend line can be interpreted as a 14.4 [0.6–28.2] minute increase per year, on average, in direct admissions over the study period. Over the same period for direct admissions, injury to ambulance arrival, pre-hospital (scene time), ambulance to ED and injury to CT time all increased significantly for this subgroup.

**Table 4:** Longitudinal regression statistics over time

	Slope	95% CI	p-value
*Injury to operating room time*			
Injury to OR (Total)	0.29	-0.06 to 0.64	0.10
Injury to OR (Direct Admissions)	0.24	0.01 to 0.47	0.04
Injury to OR (Referred from peripheral hospital)	0.28	-0.67 to 1.2	0.54
Injury to OR (Isolated head injury)	0.39	-0.25 to 1.0	0.22
Injury to OR (Polytrauma)	0.21	-0.14 to 0.56	0.23
*Direct Admissions subgroup*			
Injury to ambulance time (Direct admissions)	0.05	0.01 to 0.09	0.02
Ambulance to ED time (Direct admission)	0.06	0.004 to 0.11	0.03
Pre-hospital time (Scene Time) (Direct Admission)	0.1	0.02 to 0.17	0.01
ED to CT time (Direct Admissions)	0.02	-0.04 to 0.08	0.55
ED to OR time (Direct Admissions)	0.05	-0.15 to 0.25	0.63
Injury to CT time (Direct Admissions)	0.15	0.04 to 0.25	< 0.01
CT to OR time (Direct Admissions)	0.05	-0.13 to 0.24	0.58
*Prognosis and mortality*			
Mortality % (Total)	0.55	-2.53 to 3.63	0.70
Average GOS on discharge	0.01	-0.19 to 0.21	0.89
Average GOS on follow up	0.02	-0.18 to 0.22	0.82
Average change in GOS score	0.01	-0.02 to 0.05	0.45

**Fig. 4:**
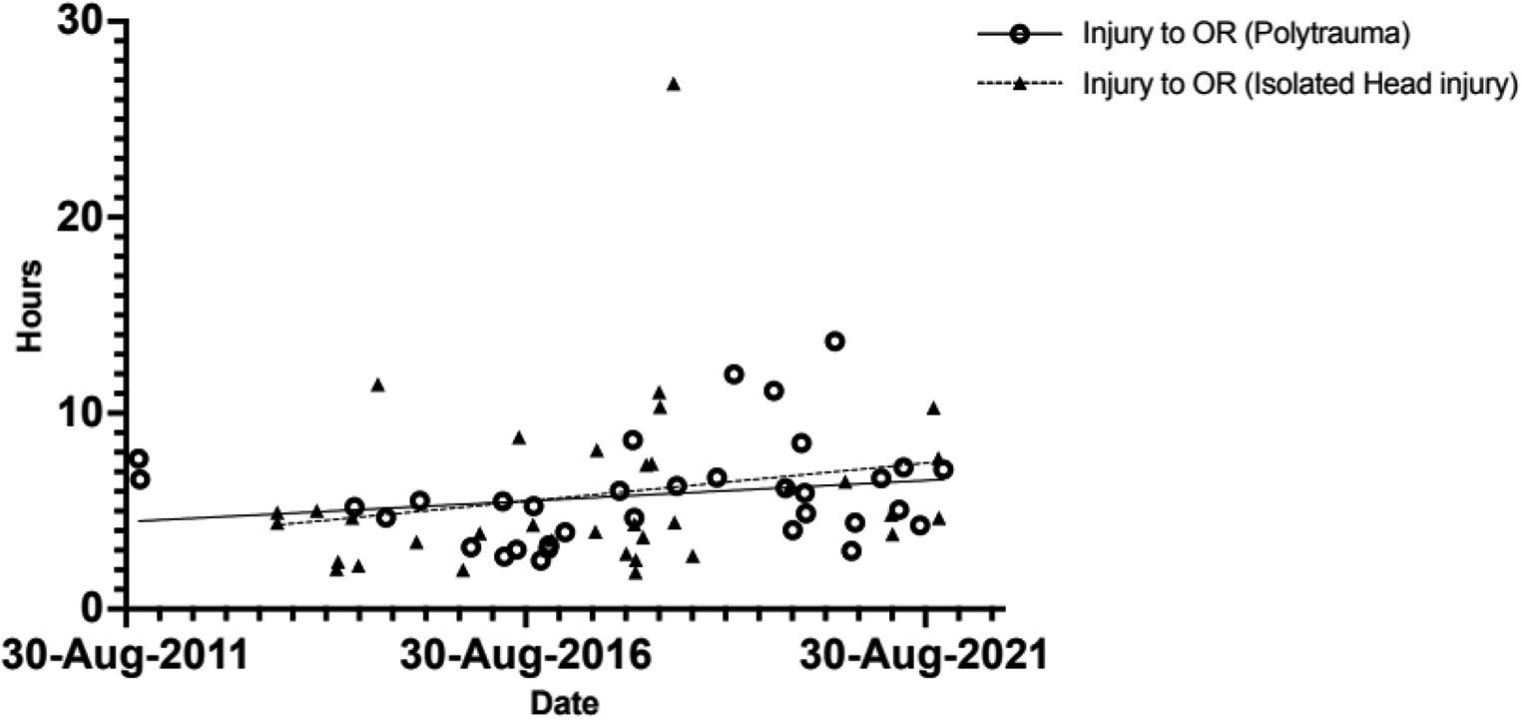
Injury to OR (Isolated head injury versus polytrauma)

Injury to OR times for referrals from peripheral hospitals, polytrauma and isolated head injuries were similarly plotted against time. The time from injury to OR was unchanged over the study period (Table 4 and Fig. 4).

Linear regression of total time spent in ED, ED arrival to CT head, ED to OR time and CT to OR time for the whole cohort and for all subgroups was not statistically significant (Table 5).

**Table 5:** Linear regression statistics for key points in ED timing data

	Whole cohort	Isolated head injury	Polytrauma	Direct admissions	Referred from peripheral hospitals
ED total time	0.02 (-0.01 to 0.16), 0.76	0.04 (-0.14 to 0.22), 0.63	-0.01 (-0.22 to 0.20), 0.94	0.06 (-0.05 to 0.19), 0.26	0.18 (-0.44 to 0.31), 0.72
ED arrival to CT head	-0.006 (-0.07 to 0.06), 0.86	0.06 (-0.02 to 0.13), 0.15	-0.05 (-0.13 to 0.04), 0.28	0.02 (-0.04 to 0.08), 0.54	-0.06 (-0.24 to 0.13), 0.53
ED to OR time	-0.01 (-0.18 to 0.15), 0.88	0.09 (-0.21 to 0.38), 0.56	-0.09 (-0.30 to 0.12	0.10 (-0.16 to 0.25), 0.65	-0.11 (-0.39 to 0.16), 0.40
CT to OR time	0.18 (-0.06 to 0.43), 0.14	0.26 (-0.11 to 0.63), 0.17	0.04 (-0.30 to 0.39), 0.79	0.05 (-0.13 to 0.24), 0.59	0.16 (-0.48 to 0.80), 0.60

Values reported in format: *slope (95% Confidence interval), p-value*

One outlier, identified via ROUT analysis with Q = 0.5%, was removed as a sensitivity analysis to see if it would affect the slope of our regression analysis. Removing the outlier did not have a significant effect on the results.

**Fig. 5:**
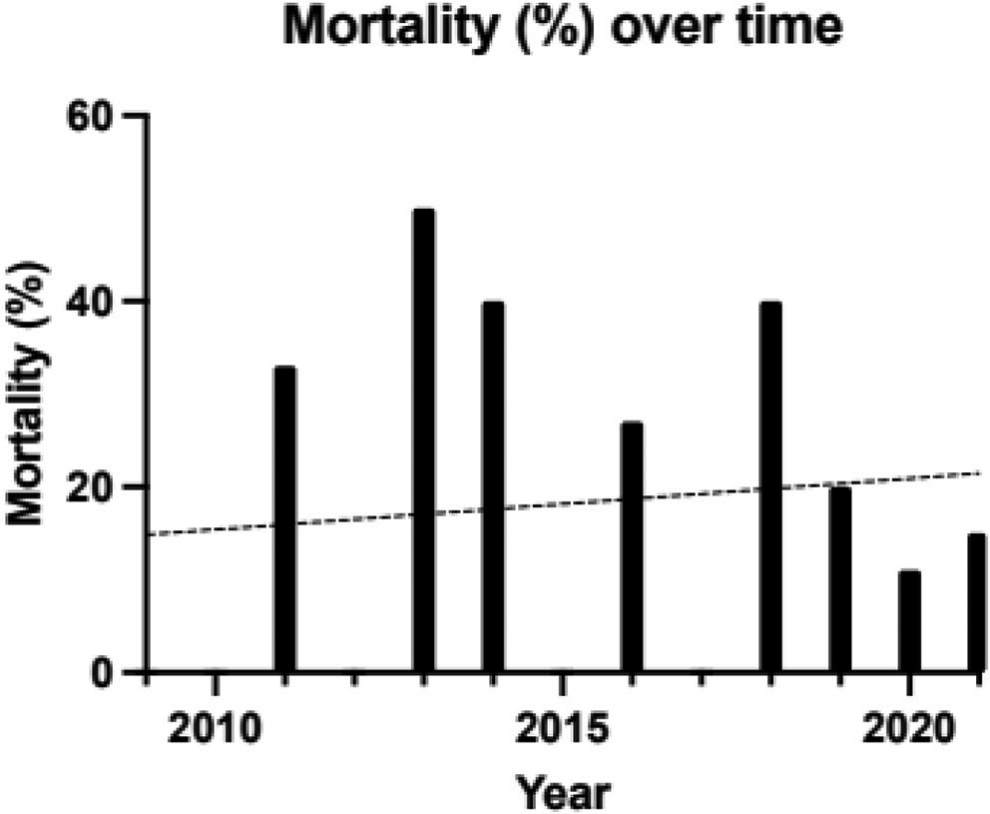
Mortality versus year

Duration of operation was similar across all groups. Mortality was lower in the secondarily referred group compared to the patients who were admitted directly, 2 (8.00%) versus 19 (35.85%), *p* < 0.001. Mortality was not different statistically between isolated head injury and polytrauma. Mortality over time did not show a statistically significant difference (Fig. 5; Table 4). Median GOS Score on discharge was lower in direct admissions compared to all other groups, *p* < 0.001. GOS score at follow up was higher in direct admissions (median 4) and those referred from peripheral hospitals (median 5), *p* < 0.001. Median GOS on discharge and follow up was unchanged for the total cohort and for isolated head injury and polytrauma patients. There was no change in average GOS on discharge (slope = 0.01, *p* = 0.89) or follow up (slope = 0.02 (-0.18 to 0.22). *p* = 0.82) over the study period (Table 4).

## Discussion

This study provides contemporary timing of surgical intervention data for patients with traumatic intracranial hematoma. Traumatic intracranial hematoma encompasses subdural, epidural, intraparenchymal and combinations of these types of intracranial hematoma. As the patient’s included in our study were of high acuity requiring urgent neurosurgical decompression regardless of individual pathology, we treated all these pathologies as one entity. We collected data on other key points to look for timing areas which could be reduced with hospital or pre-hospital policy. Only one other study has broken down the injury to OR time thoroughly prior to our study [11], some studies have broken down their timing data focusing on the interval between CT head to OR but have not explored other subintervals [15, 16]. We assessed prognosis of our cohort in terms of mortality and neurological outcome, by way of the Glasgow outcome score. A unique finding for our study is that injury to operating room time for direct admissions to our hospital has increased over time. The time to definitive operative intervention in direct admissions increased by 14.4 min, on average, every year. Despite this, there was no appreciable change in mortality over the study period. One possible explanation is that mortality is primarily governed by the severity of primary injury whereas time to operative intervention is aimed at reducing secondary injury. Our median injury to operating room time for the total cohort fit within the pre-reported range in the literature of 3–7.1 h. Another interesting finding was shorter ED to OR times in referred patients as compared to direct admission. Referred patient’s had CT scans at peripheral hospitals with a known indication to proceed to OR. As such, plans to ready the OR and personnel required could be made while the referred patient was in transit to our institution which explains the shorter ED to OR times in this cohort.

The median time spent in the ED was 1.6 h in our cohort and represents a substantial proportion of the total time between injury and OR. One potential explanation for delays to OR over this period is strain on health resources over a similar period. Between 2011 and 2022 ED presentations at our institution increased 61% for triage category 1 patients and 102% for triage category 2 patients [17]. It is not clear whether this would account for delays to operative intervention however as appropriate operating room staffing and availability as well as prioritization of urgent surgical cases would counteract this increase.

Another potential explanation is longer resuscitation and investigation in the emergency department prior to transfer to OR. Investigation and correction of the coagulation system often takes place in the ED. Correction of coagulation and administration of correction products was outside the scope of this study as all patients at our institution routinely receive hemostatic resuscitation during initial resuscitation efforts and during induction for anesthesia. A factor that has been explored in previous studies is the use of tranexamic acid (TXA). TXA administration for ICH has been shown to reduce hematoma expansion however it has not been shown to affect neurological outcome or survival in large scale trials or subsequent systematic reviews including them [18, 19]. It is not routinely used in the resuscitation of severe head injury at our institution. Further, our study aimed to include only patients with high acuity that needed life-saving decompression of TICH which would have taken precedence over correction of coagulopathy for the reasons set out above. To explore this, we conducted a regression analysis of important times between events in the ED. We did not find any statistically significant increase in the average times of total time spent in ED, ED arrival to CT head, ED to OR time or CT to OR time over the study period.

Another important modifiable factor is injury to ED time. During the Oct-Dec quarter in 2020, NSW Ambulance response times to level P1a (the highest acuity emergencies) emergencies were 8 min whilst P1 (second highest level of acuity) emergencies were 12 min for the local government area that the hospital services [20]. The median response time for our cohort was 19.2 min. Interestingly, our cohorts median initial GCS of 8 dropped to a median of 3 by the time they reached the hospital’s ED. It is plausible that the increased ambulance response time seen in our cohort could be since some head injured patients seem well immediately after an injury however can rapidly deteriorate with extension of hematoma and loss of compensatory reserve. A likely explanation of this decrease is a higher rate of pre-hospital intubation. Prehospital intubated patient’s GCS decreased from 7 to 3 whilst non-intubated patients GCS from 13 to 10. Future prospective studies could look at the reasons for deterioration in this cohort to see if the decrease in GCS between ambulance arrival and arrival at ED is due to sedation and intubation or from brain injury deterioration.

ED to CT is considered a factor which could be improved with better hospital systems and processes and therefore has potential for significant improvement in removing delays to the operating room. The median ED to CT time in our study was 1.20 h (IQR 0.91–1.74). As a percentage of median total ED time, ED to CT represented 75%. This is a crucial area for improvement as reducing the interval between patient and arrival and CT allows neurosurgeons to make important management decisions quicker. Our institution’s emergency department is some 30 years old without an integrated radiology department meaning patients need to be transported some distance to receive CT head scans which further delays neurosurgical diagnosis. Surprisingly, data in the literature is lacking regarding ED admission time to CT head with many studies reporting data as injury to CT. Two large studies looking at TBI patients progressing to operative intervention found that delay to initial CT head was associated with longer times to operative decompression [21, 22]. 

A limitation of our paper is the small sample size due to the relatively rare event and retrospective nature of study. Due to the smaller sample size a longer study period was needed. Although no large change to brain injury management occurred during this time frame, smaller incremental changes to our trauma system may indirectly affect severe brain injury management. Regardless of the examined study population, this is still the highest volume trauma center in our state with the largest number of Level-3 trauma center referring base. The literature is divided on the impact that delay to decompression has on functional outcome and mortality. Criticisms of these studies have been based on their inclusion criteria, specifically of their inclusion of all TBI patients or inclusion of CSF diversion therapies as well as craniotomy or craniectomy. We aimed to provide data on only acute patients requiring emergent operative intervention for hematoma evacuation via craniotomy or craniectomy, which in part explains our small sample size.

Our study reports on timing data in the most granular way possible which can be useful for other trauma centers and trauma systems to benchmark against. This approach can provide direct comparisons among institutions rather than timing data as less than or greater than pre-determined cut off values i.e., less than or greater 4 h [23–28]. The detailed breakdown of injury to surgery times would allow more detailed analysis to determine where room for improvement may lie.

There is room for improvement in minimizing delays to OR in this cohort of TICH patients. We have provided contemporary timing data including median values of key timing intervals between injury and OR. This study informed our institution to act urgently on the increasing times to surgery in TICH patients and suggests that other trauma systems should critically evaluate their results as well. Our reporting standards and reported outcomes could be used to validate other trauma systems more broadly.
